# Surface Enhanced Raman Spectroscopy for In-Field Detection of Pesticides: A Test on Dimethoate Residues in Water and on Olive Leaves

**DOI:** 10.3390/molecules24020292

**Published:** 2019-01-15

**Authors:** Lorenzo Tognaccini, Marilena Ricci, Cristina Gellini, Alessandro Feis, Giulietta Smulevich, Maurizio Becucci

**Affiliations:** 1 Department of Chemistry ‘UgoSchiff’, Università degli Studi di Firenze, via della Lastruccia 3-13, 50019 Sesto Fiorentino (Fi), Italy; lorenzo.tognaccini@unifi.it (L.T.); marilena.ricci@unifi.it (M.R.); cristina.gellini@unifi.it (C.G.); alessandro.feis@unifi.it (A.F.); 2 Department of Photonics, St. Petersburg Electrotechnical University, Ul. Prof. Popova, St. Petersburg 197376, Russia; 3 European Laboratory for Non-Linear Spectroscopy—LENS, via N. Carrara 1, 50019 Sesto Fiorentino (Fi), Italy

**Keywords:** dimethoate, pesticides, SERS, olive, portable microRaman

## Abstract

Dimethoate (DMT) is an organophosphate insecticide commonly used to protect fruit trees and in particular olive trees. Since it is highly water-soluble, its use on olive trees is considered quite safe, because it flows away in the residual water during the oil extraction process. However, its use is strictly regulated, specially on organic cultures. The organic production chain certification is not trivial, since DMT rapidly degrades to omethoate (OMT) and both disappear in about two months. Therefore, simple, sensitive, cost-effective and accurate methods for the determination of dimethoate, possibly suitable for in-field application, can be of great interest. In this work, a quick screening method, possibly useful for organic cultures certification will be presented. DMT and OMT in water and on olive leaves have been detected by surface enhanced Raman spectroscopy (SERS) using portable instrumentations. On leaves, the SERS signals were measured with a reasonably good S/N ratio, allowing us to detect DMT at a concentration up to two orders of magnitude lower than the one usually recommended for in-field treatments. Moreover, detailed information on the DMT distribution on the leaves has been obtained by Raman line- (or area-) scanning experiments.

## 1. Introduction

**Figure 1:**
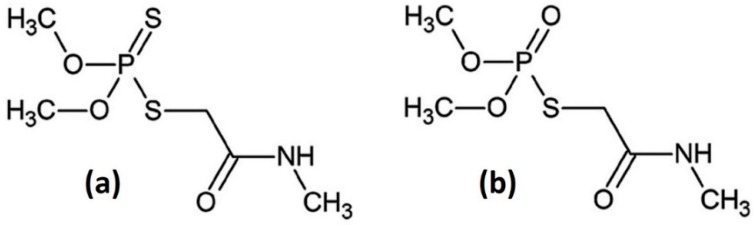
Schematic structures of DMT (**a**) and OMT (**b**).

The balance between large-scale, cost-effective production in agriculture and the public demand of healthyfood is actively pursued. Therefore, a continuous effort is made for the sustainable use of pesticides. At present, the use of pesticides is strictly regulated in many countries. Their field of application, dose and allowed residual limit are defined by public regulatory authorities and periodically revised. Different countries have specific norms concerning the allowed DMT use and dose. We will refer only to the EU regulation, however very similar rules are enforced in many other countries. Moreover, public awareness is very high and organic food is getting with time a larger and larger share of the market with a constant increase of the number of organic farms. Certification for organic food production is not trivial as some pesticides have a reduced persistence on fruits and plants. That is very good for the final quality of the products but, at the same time, it makes extremely difficult to certify the full production chain. The available analytical methods with very high sensitivity and general applicability for pesticides are mostly based on extraction and chromatographic method. Efficient routines (QuEChERS) have been devised allowing sample cleanup and multiresidue analysis of pesticides, fully responding to the present norms [1,2]. However these methods are time consuming requiring many steps (sampling, transport to the laboratory, storage of the sample, analysis), and an advanced sample manipulation including the use of different solvents and reagents. Therefore, simple analytical methods, possibly suitable for in-field application can be of large interest. They could be used for targeted analysis of selected pesticides, thanks to the knowledge of specific problems of the different cultivars and local environmental conditions that make more likely the presence of specific pests in the field. As an example, the olive fly (*Bactroceraoleae*) is probably the most important pest affecting olives in some regions (typically the Mediterranean basin and South Africa and, more recently, California). Its presence is most relevant in areas and periods characterized by higher humidity and relatively lower temperatures. A widespread pesticide used for the olive fly control is based on dimethoate (DMT) as active substance [3]. DMT is an organophosphate insecticide and acaricide used since the ‘50s. It is degraded relatively rapidly following different pathways [4]. DMT, whose structure is depicted in Figure 1, is converted into its oxygenated analogous omethoate (OMT, see also Figure 1) on the leaf surfaces while the absorbed DMT is decomposed following different enzymatic reaction pathways. This is the reason for definition of regulatory limits often given as the sum of DMT and OMT content [5]. However, more recently different regulatory limits have been defined for the two pesticides [6].

DMT is suggested for treatments on olive plants as a water solution at 10^−2^–10^−3^ M concentration range, according to the technical datasheet from the producer Cheminova AS for the different treatment strategies (80–625 mL of the industrial formulation containing 40% DMT in weight per 100 L of the final solution). It has a deadline deficiency (interval between treatment and use of product) of 28 days. Its persistence (either as DMT or OMT) on the surface of the olive fruit is up to 10% on the 4 weeks timescale [4,7,8]. The presence of DMT on olives for oil production is allowed to the maximum limit of 3 mg/Kg (OMT 1.5 mg/Kg) [6].

DMT is also found in water bodies following its use in field. DMT is conveniently used in the olive farms as it is highly water soluble and its transfer from olive to oil during oil production is low (about 30%) [8]. That is an interesting property for the olive oil production but poses severe issues on the treatment of the residues from olive oil production. The regulatory framework for water policies is set by international laws [9] but national limits apply for specific cases. For instance, in Italy DMT contaminated water can be sent to the sewage system if total organophosphorus pesticides concentration, which includes DMT, is below 0.1 mg/L [10]. The same limit applies for the direct discharge in surface water bodies. DMT is used not only to support olive production but also in many productions, including citrus, lettuce, tomatoes, onions, and others. Therefore, the problem is quite general and the availability of simple and effective methods for its in-field identification could be of extreme interest for the public health and for the appropriate certification of organic produce.

Recently, it has been shown that surface enhanced Raman spectroscopy (SERS) is an effective method for DMT detection both in water solution and on solid surfaces [11]. It is well known that Raman scattering intensity can increase enormously in presence of nanostructured metal surfaces, typically silver or gold [12,13,14]. If the molecule under investigation is efficiently adsorbed on the metal surface, it can experience an extremely high local electric field due to the nanostructured surface topology and the resonant excitation of localized surface plasmons. Furthermore, chemical processes can affect the adsorbed molecules and modulate its polarizability, which is responsible for the scattering process itself.

In recent years, different examples of application of SERS spectroscopy for analytical purposes and more specifically for detection of pesticides on vegetables have been presented (see for instance refs. [15,16] for recent reviews). Few examples are reported in the literature for the identification of different pesticides by direct application of silver nanoparticles (AgNPs) on fruit or vegetable skin [17,18,19,20,21,22,23,24,25]. These experiments are carried out using confocal microscopes coupled to laboratory equipment. Very recently, this approach was used for measuring the OMT distribution on apples and peaches [26,27]. The use of portable equipment is still a challenge for identification of pesticides directly on the vegetables surface, without intermediate steps [28].

In this paper, we report new results useful for identification of olive leaves treated with DMT by means of SERS. We have determined a limit of detection for DMT in water and identified the presence of DMT on olive leaves using portable instrumentation. Therefore, this work provides a basis for in-field DMT detection in olive cultivations and on-line monitoring process for the DMT content in wastewaters from olive oil production plants.

## 2. Results and Discussion

**Figure 2:**
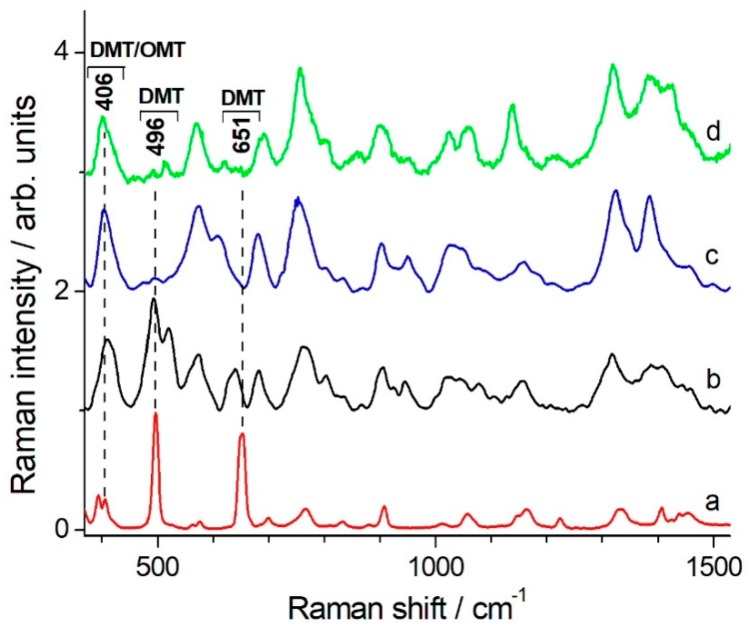
Raman spectrum of solid DMT (**a**), SERS spectra of 10^−4^ M DMT measured immediately after sample preparation (**b**) or 15 min later(**c**), and OMT SERS spectrum (**d**) (adapted from Guerrini et al. [11]). Experimental details: (**a**) measured on microRaman Renishaw RM2000 spectrometer with 10s accumulation time and 3 averages; (**b**) and (**c**) measured on BWTek portable spectrometer with 50s accumulation time and 4 averages. Spectra are baseline corrected. The wavenumbers of the characteristic Raman bands of DMT and OMT discussed in the text are indicated.

The DMT and OMT Raman and SERS spectra have been discussed in details (including bands assignment) by Guerrini et al. [9]. The authors showed that the SERS spectra of DMT on AgNPs could not be simply related to the Raman spectrum of solid DMT, the intensity and frequency variations being much larger than usual. They suggested that AgNPs promote DMT hydrolysis to omethoate (OMT), a reaction that is known to occur also through thermal or photocatalytic mechanisms. In Figure 2 we show the comparison of the DMT (solid) Raman spectrum (Figure 2a) and the OMT SERS spectrum (Figure 2d) with SERS spectra of DMT in solution taken at different delay times after preparation. 

The SERS spectrum of a freshly made solution (Figure 2b) still exhibits the strong band at 496 cm^−1^ that dominates the solid DMT Raman spectrum. This band almost disappears 15 min after sample preparation and the spectrum (Figure 2c) becomes practically coincident with the OMT SERS spectrum. Therefore, these new results strongly support the hypothesis of a rapid DMT decomposition to OMT, promoted by AgNPs, in water solution.

Once the Raman/SERS spectroscopic signature of DMT was firmly established, our aim was to identify the most suitable experimental conditions for a high sensitivity DMT detection by SERS methods. Also, we have determined the limit of detection for DMT in solution under our best operative conditions and we have established a protocol for DMT identification when it is deposited and dried on solid surfaces. Our experiments have been carried with either laboratory or portable Raman setups.

AgNPs, synthesized according to the Lee-Meisel method [29], were selected as SERS active substrate. The plasmonic properties of the AgNPs were checked measuring their UV-VIS attenuance spectrum (Figure S1 in the Supplementary Information). The maximum was around 418 nm, corresponding to a most probable diameter of about 40 nm [30]. The TEM image of the AgNPs is shown in the Supplementary information, Figure S2. 

SERS spectra of DMT (10^−4^ M in the AgNPs colloidal dispersion) were obtained using excitation wavelengths at 458, 532, 633, 785, and 1064 nm. With the shorter wavelengths (458 and 532 nm), the intensity of the SERS signal was very weak (data not shown). Therefore, we worked with long-wavelength laser sources. In fact, a partial NPs aggregation induced by ions in solution caused the formation of strongly SERS-active aggregates of nanoparticles characterized by a red-shifted plasmonic band [13]. This aggregation can be followed spectroscopically by checking the changes of the UV-VIS attenuance spectrum with the concentration of these anions. Both a decrease of the peak at 418 nm and an increase of the longer wavelength wing of the signal can be clearly observed (see Supplementary Information, Figure S1). A 100 mM KNO_3_ concentration in solution completely destabilizes the Ag colloidal dispersion. Instead, at 40 mM KNO_3_ concentration the colloidal dispersion is still stable during the measurements and a strong aggregation is spectroscopically observed. As a further step, we have measured the relative SERS signal enhancement for DMT with 785 and 1064 nm excitation in the presence of 10% (*v*/*v*) ethanol, which is not adsorbed on AgNPs and does not destabilize the colloidal dispersion. Therefore, the strong Raman peak of ethanol at 880 cm^−1^ can be used as internal standard for intensity calibration of the DMT SERS signals. As shown in Supplementary Information (Figure S3), we observed a signal enhancement approximately 2 times higher in the DMT spectrum taken with the 785 nm excitation as compared to the 1064 nm excitation. Therefore, we used the 785 nm laser excitation for the majority of the experiments. This is an advisable choice also because many portable Raman spectrometers working in this spectral range are commercially available.

The SERS analytical enhancement factor observed in our experiments with 1064 nm excitation is in the order of 10^3^, evaluated from the ratio of the strongest DMT bands at 496 and 651 cm^−1^, measured in a 10^−2^M DMT solution in water (solubility limit) and in the SERS spectrum obtained immediately after sample preparation. This value should be taken as a lower limit for the SERS analytical enhancement factor due to the rapid DMT decomposition to OMT in the SERS samples. The DMT Raman spectrum in solution is shown in the Supplementary Information, Figure S4.

**Figure 3:**
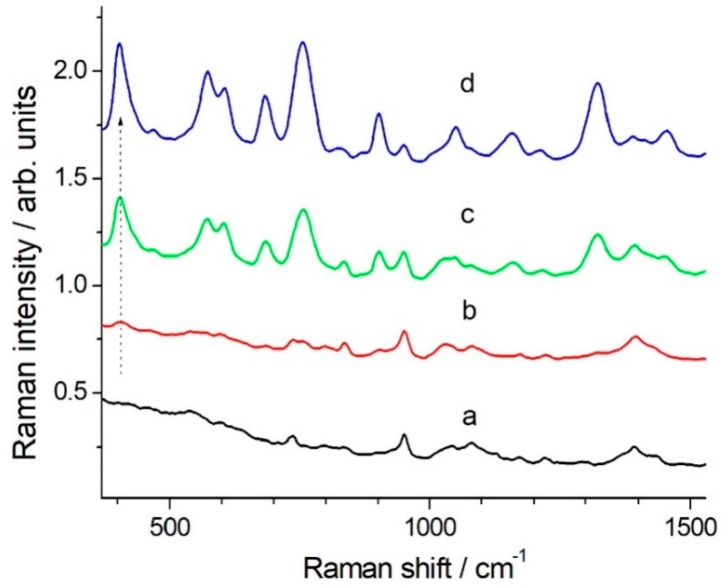
SERS spectra (785 nm excitation, BWTek portable Raman spectrometer) for different nominal DMT concentration: 0.0 (only AgNPs in water) (**a**), 1 × 10^−6^ (**b**), 5 × 10^−6^ (**c**), 1 × 10^−5^ M (**d**). Spectra are vertically shifted to improve data readability. The arrow points to the 406 cm^−1^ band used for DMT quantification.

**Figure 4:**
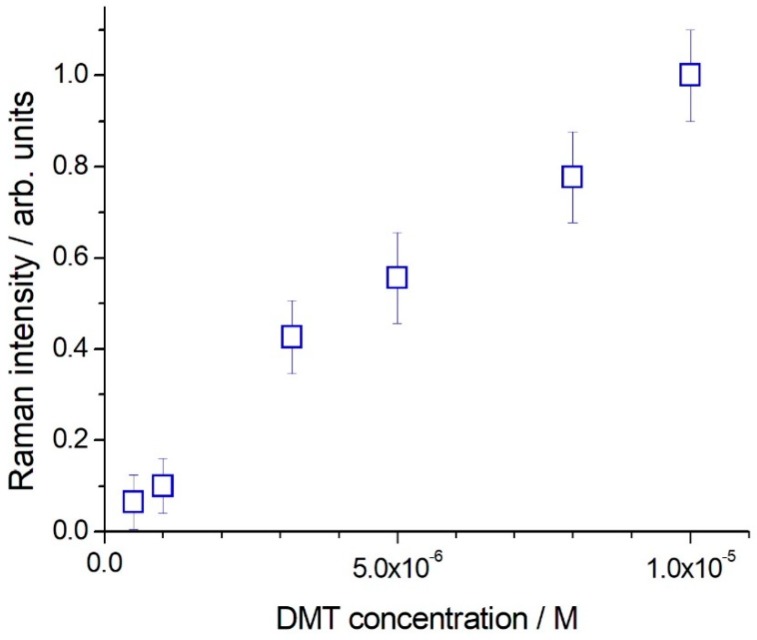
SERS signal (785 nm excitation, BWTek portable Raman spectrometer) of the band at 406 cm^−1^ as a function of DMT nominal concentration, measured on three sets of samples. The standard deviation for each data point is reported.

Application of SERS for quantitative analysis is quite difficult [31,32]. Also, the clear definition of the limit of detection (LOD) for an analyte in Raman/SERS spectroscopy is not straightforward. The signal-to-noise ratio depends on optical layout, characteristics of the detector, available laser power, integration time and number of averaged spectra and quality of the SERS active substrate, among the other parameters. However, for a practical application of the SERS method we must try to provide some indication: Figure 3 shows typical spectra, which were obtained with an integration time of 100 s, 5 averages, laser wavelength 785 nm, 20 mW at the sample using the portable system. The most suitable band for a quantitative data evaluation is that at 406 cm^−1^, a single peak standing out of a flat background. The peak appears to be common to DMT and OMT [11]. By the use of that reporting signal, the DMT detection limit is determined as low as 5 × 10^−7^ M, corresponding to a signal to noise ratio equal to 3 in the peak height determination. A good linearity for the SERS signal at 406 cm^−1^ as a function of the DMT concentration was obtained in the range 5 × 10^−7^–1 × 10^−5^ M (see Figure 4).

In order to test the possibility to perform SERS experiment *in-situ* to identify DMT on olive leaves, we have measured the Raman/SERS signal originated from olive leaves obtained with the 785 nm excitation using a Raman microscope. No relevant Raman signal is originated from the bare leaf (either front or back surface), only a modest luminescence background is present. Upon deposition of AgNPs on the leaf surface, no relevant SERS signal is observed and the luminescence is slightly quenched.

As a first test carried out with laboratory equipment, we prepared mock-ups by spraying DMT and AgNPs on a glass slide and we measured their SERS spectra. In details, we sprayed on a glass slide a 10^−2^ M solution of DMT with aggregated AgNPs, similar to those used for the tests in solution described above. The concentration of DMT corresponds to that recommended for in-field treatments. The sprayer produced a good aerosol that covered uniformly the surface with 20–100 μm diameter droplets. SERS spectra with very good S/N ratio and very similar to those measured on DMT solutions (see Supplementary Information, Figure S5) have been obtained.

**Figure 5:**
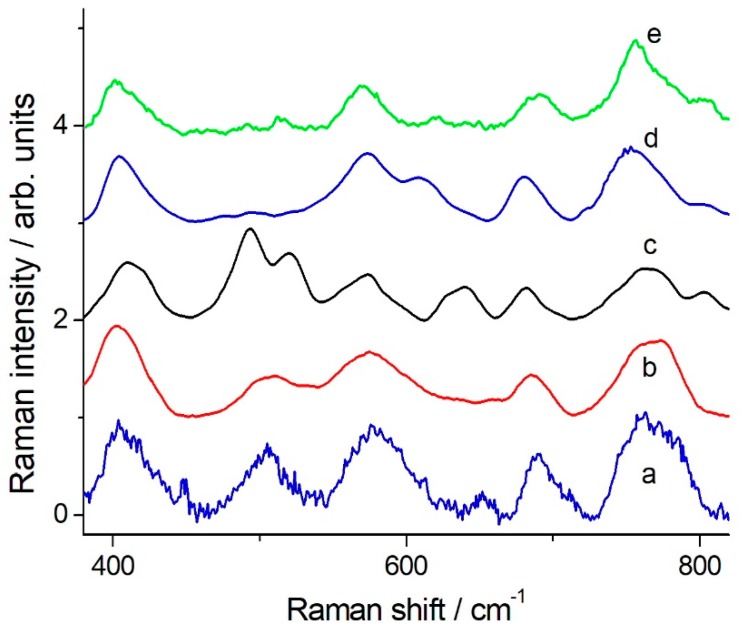
Comparison between the SERS spectra measured on olive leavesDMT spikedand SERS spectra of the standard materials in solution. (**a**) SERS spectrum measured with laboratory equipment on the olive leaf spiked with 10^−4^ M DMT. (**b**) SERS spectrum measured with portable equipment on the olive leaf spiked with 10^−2^ M DMT. (**c**) SERS spectrum of 10^−4^ M DMT measured immediately after sample preparation. (**d**) SERS spectrum of 10^−4^ M DMT measured 15 min after sample preparation. (**e**) OMT SERS spectrum (adapted from Guerrini et al. [11]). Experimental conditions: 785 nm laser excitation; (**a**) measured with Renishaw RM2000 microRaman spectrometers; (**b**,**c**,**d**) measured with a BWTek portable Raman spectrometer; (**a**) 2 mW at the sample, 20x objective, 10 s integration time, 20 averages; (**b**) 2.5 mW at the sample, 40x microscope objective, 10 s integration time, 10 averages; (**c**,**d**) 20 mW at the sample, macro Raman fiber probe, 50s integration time, 4 averages. All spectra are baseline corrected.

Furthermore, still using laboratory equipment, DMT solutions (10^−2^ –10^−4^ M) were sprayed on olive leaves, later the colloidal dispersion of AgNPs was applied. We let the samples dry in air and then we measured the SERS spectra in many different points with a Raman microscope. The SERS signal was detected with a reasonably good S/N ratio even for samples spiked with DMT 10^−4^ M, as shown in Figure 5a. In this case the spectrum is intermediate between that of OMT and DMT as the peak around 500 cm^−1^, characteristic of DMT, is present (or even strong and comparable to all other major features present that are typical of the OMT SERS spectrum [11]), suggesting that only a partial DMT hydrolysis has taken place on the leaf surface.

**Figure 6:**
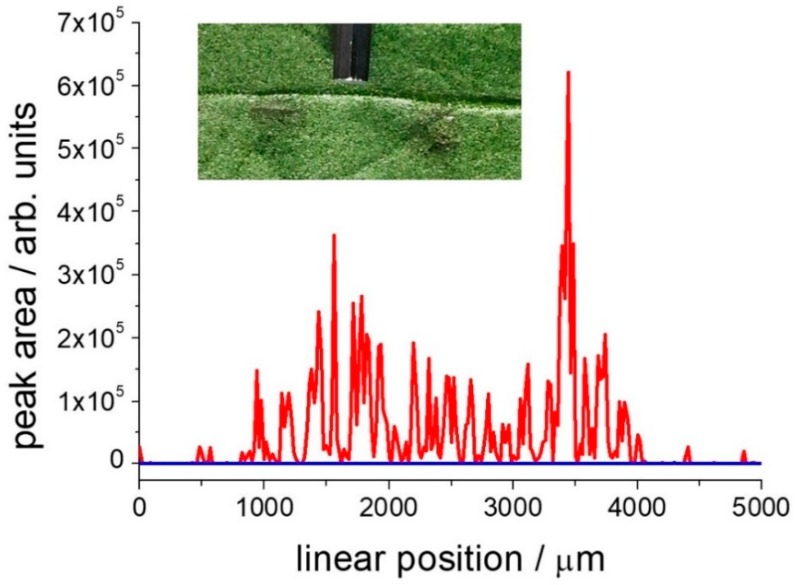
Plot of the 406 cm^−1^ peak area in a 5 mm line-scan (20 μm step) passing across a 3 mm diameter area treated with the AgNPs solution, either DMT treated or not. The flat, blue line refers to the DMT-free leaf surface; the red line refers to the DMT-treated leaf surface. The inset show a picture of the leaf and the two measured areas (DMT treated and not), possibly identified as grey circles due to the presence of AgNPs. We placed the tip of a 2 mm hex key in between the two spots to set the scale of the image. Data obtained with the Renishaw RM2000 Raman microscope.

Detailed information on the DMT distribution on the leaves could be obtained by Raman line- (or area-) scanning experiments that we performed by placing the sample on a computer controlled motorized micrometric table and acquiring the spectrum in different points. As discussed above, under these experimental conditions DMT rapidly transforms to OMT. However, for the purpose of organic grown cultivation methods, what is relevant is the detection of DMT or its derivatives, not its exact quantification. Since the 406 cm^−1^ Raman band does not show any major overlap with other spectral components, only a small section of the spectrum around this band was selected for numerical elaboration (typically between 350 and 450 cm^−1^). It was possible to fit the spectrum by using only a linear baseline and one single band with a Lorentzian profile, total five free parameters, with appropriate constrains for the band center position and bandwidth. Automatic fitting routines that operate on larger spectral regions, including many bands, do not give reliable results due to the large number of parameters needed. In Figure 6 we present the intensity signal of the 406 cm^−1^ band for line-scans made across a leaf area treated with 2 μL of AgNPs colloidal dispersion, either clean or spiked with 1 μL of 10^−3^M DMT solution. The experiment gives virtually zero signal on the clean leaf surface or in that covered with AgNPs only. Conversely, it provides a clear signal with 10% standard error in the leaf region treated with DMT/AgNPs (visually measured as large as 3 mm). The large fluctuations of the signal intensity in the leaf spiked area are probably due to the ratio between the small sampling area accessed in each point by the confocal Renishaw Raman microscope and the scale of the sample spatial variance, that is related both to the leaf morphology and the uniformity of surface coverage with DMT/AgNPs. Images of olive leaves, clean or treated with AgNPs, are shown in the Supplementary Information (Figure S6).

We have also measured the SERS signal on a DMT treated olive leaf, prepared as described above, by using the portable BWTek microRaman setup. The DMT SERS spectrum on the leaf surface was obtained as the difference between the signals measured on the spiked and clean areas (the full set of data is reported in the Supplementary Information, Figure S7). The leaf area treated with DMT was spiked with 1 μL of DMT solution 10^−2^ M followed, on the dried leaf, by 2 μL of AgNPs colloidal solution. The SERS spectrum obtained with 2.5 mW laser power showed a very good S/N ratio (Figure 5b), while a laser power above 10 mW resulted in the drop of the SERS signal and the raise of a strong background, possible indication of sample heating and desorption of the analyte from the AgNPs.The SERS spectrum measured on the leaf surface (Figure 5b) closely matches the one measured on freshly made DMT solutions (Figure 5c), suggesting the occurrence of a limited DMT hydrolysis.

**Figure 7:**
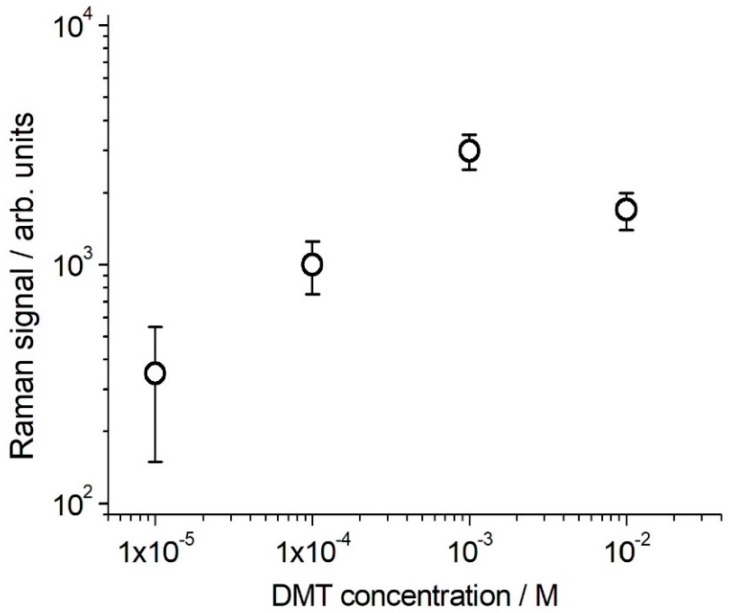
DMT determination with the BWTek portable microRaman spectrometer on olive leaves. Logarithmic plot of the SERS signal (406 cm^−1^ band area) vs. DMT concentration. Conditions: 40× objective, 785 nm excitation wavelength, 2.5 mW laser power on the sample, 10 s integration time and 10 averages.

Finally, we carried out a test for the detection of DMT on the olive leaves spiked with DMT water solutions at different concentration, ranging from 10^−2^ to 10^−5^ M, by using the portable micro-Raman spectrometer. This measurement is representative of the in-field DMT identification at later time after treatment (it is commonly applied as a 10^−2^–10^−3^ M solution and its persistence on the olive fruit surface is possibly as large as 10% after 4 weeks). Figure 7 shows the results, in a logarithmic plot, based on the average of different determinations carried out on the leaf surface DMT spiked (either with 1 μL droplet of DMT solution or sprayed with the same solution, two replicas each) and AgNPs coated (2 μL droplet). Our data show a good sensitivity in the 10^−4^–10^−2^ M DMT concentration range, suggesting an effectiveness of the method for DMT SERS detection on leaves up to 4–8 weeks after treatment, according to the recommended treatment strategies. The non-linearity shown by the plot for high DMT contents is commonly observed in SERS quantitative applications that span a large concentration range [33]. The SERS signal depends on the interaction of the analyte with the AgNPs, a process influenced by many parameters, including the analyte concentration itself.

## 3. Materials and Methods

DMT (analytical standard quality) was purchased from Merck/Sigma-Aldrich (Darmstat, D.) and was used without further purification. Water used for preparation of the different solutions was LC-MS chromatography grade, obtained from Merck/Sigma-Aldrich. Silver nanoparticles were prepared as colloidal dispersions according to the standard Lee-Meisel method [29]. Their extinction spectrum in the visible range was used as a probe of both dimensions distribution and aggregation [30,34].

SERS spectra in solution in standard 10 mm squared base quartz cuvette were measured in backscattering geometry either using a Bruker FT-Raman spectrometer (Bruker, Billerica, MA, USA, 1064 nm excitation, 20–300 mW on the sample) or using a portable Raman spectrometer. The latter was assembled using a 785 nm narrow-band diode laser as light source, coupled to a Raman fiber probe (InPhotonics, Norwood, MA, USA,) delivering 20 mW laser power at sample. The scattered light was fed through the fiber to an Exemplar Pro spectrometer (B&W Tek Inc., Metrohm, Herisau, Switzerland) with deep-cooled CCD detector. Solutions with concentrations ranging from 10^−2^ to 10^−7^ M were prepared by adding to 900 μL of the colloidal dispersion both 40 μL of a 1M solution of KNO_3_ (as aggregant) and 100 μL of a standard DMT solution in water. SERS spectra were measured almost immediately after sample preparation. Preliminary tests for assessment of the best excitation wavelength were made also on a table-top Raman spectrometer consisting of a SP150 monochromator (Acton Optics, Acton, MA, USA) with a Pixis CCD detector (Princeton Instruments, Trenton, NJ, USA) and excitation laser sources at 457, 532 and 632 nm). Due to the spectral dependence of the SERS enhancement factor on the excitation wavelength, significant SERS spectra were obtained only for long wavelength excitation and the data in the Results section refer to these experimental conditions. For a more refined evaluation of the SERS enhancement factor ratio between 785 and 1064 nm excitation, the SERS measurements were repeated adding ethanol as internal standard (10% in volume).

SERS spectra on DMT solutions dried on surfaces (glass slide or olive leaves) were measured on samples prepared by using two different procedures. In the first, the samples described above (10^−2^–10^−4^ M DMT solutions in presence of AgNPs) were sprayed on the glass/leaves surface. In the second, more realistic samples were prepared by deposition of 1 μL of 10^−2^ M DMT water solution followed, after 1 h, by deposition on the same spot of 2 μL drop of colloidal dispersion aggregated by KNO_3_ (see above). Laboratory measurements were carried out on a RM2000 Raman microscope (Renishaw, Wotton-under-Edge, UK) equipped with a 20× microscope objective, on a 3 μm diameter spot. Raman mapping was performed by moving the sample on a motorized micrometric table, acquiring the spectrum in different points and plotting the intensity of a representative band as a function of the sampling point position. In order to assess feasibility of in-field measurements, final tests were performed, still in laboratory, by using a portable microscope assembly built using BWTek spectrometer with the BAC151B micro-sampling system. It allows to obtain with the 40× microscope objective a laser spot about 50 μm diameter in the focal plane, much larger than the Renishaw laboratory Raman microscope.

## 4. Conclusions

The application of SERS for in-field detection of olive cultures treated with DMT allows us to reach a good level of sensitivity by direct measurements on olive leaves. Given the usual initial treatment dose and a DMT decay rate of about 90% in 4 weeks, DMT treatment could be detected up to 1–2 months after its use. The period of DMT application in-field can be predicted with reasonable accuracy given the local weather and the spread of the pest in the local area. Therefore, this method can be useful to assess the effective DMT use in field, at least at the level of pre-screening tests.

The DMT detection in water samples could be obtained quantitatively, thanks to a good calibration or the use of the well-known Standard Addition Method. We observed a linear dependence of the SERS signal of DMT in the range 5 × 10^−7^–1 × 10^−5^ M. This sensitivity could be useful for monitoring waste water in olive oil production sites.

## Supplementary Materials

The following are available online at http://www.mdpi.com/1420-3049/24/2/292/s1, Figure S1: The attenuance spectrum (2 mm path-length) of the colloidal dispersion with AgNPs from synthesis and with different concentration of KNO_3_ (aggregant), Figure S2: TEM image of partly aggregated AgNPs deposited on a clean surface, Figure S3: Raman spectrum of ethanol taken with the 785 nm excitation (a), and SERS spectra of 10^−4^ M DMT taken with the 1064 (b) and 785 nm (c) excitation, in presence of 10% ethanol. The arrow points to the ethanol strong band (880 cm^−1^) used to evaluate the relative SERS enhancement factors. Spectra are baseline corrected, Figure S4: Raman spectrum of DMT 10^−2^ M in water (Bruker FT-Raman spectrometer, 1064 nm, 300 mW, 5000 averages, background subtracted), Figure S5: Comparison of solid DMT Raman spectrum (a) with SERS spectra of 10^−4^ M DMT (b) and of the same solution sprayed on a glass surface and dried (c). (a) and (c) measured on microRaman Renishaw RM2000 spectrometer;(b) measured on BWTek portable spectrometer, Figure S6: Microscope bright field images of clean and AgNPs treated olive leaves. (a), (b) clean olive leaf (back side) observed with 10x and 100x objectives, reflection mode; (c) clean leaf (back side), 100x objective, transmission mode; (d) olive leaf treated on the back side with aggregated AgNPs, 100x objective, transmission mode; Figure S7: Measurements with the BWTek portable micro-Raman spectrometer: (a) SERS spectrum of the DMT treated area of an olive leaf (10^−2^ M DMT), (b) Raman spectrum of the clean leaf, and (c) their difference spectrum.Experimental details: 40× objective, 785 nm excitation wavelength, 2.5 mW laser power on the sample, 10 s integration time and 10 averages. The inset shows the difference spectrum (background subtracted) that in Figure 5 is compared to those obtained from DMT/OMT solutions.

## Abbreviations

AgNPs	Silver nanoparticles
CCD	Charge coupled device
DMT	Dimethoate
LOD	Limit of detection
OMT	Omethoate
SERS	Surface Enhanced Raman Spectroscopy
S/N	Signal to noise
TEM	Transmission electron microscopy
