# Supplementary material for: Surface Enhanced Raman Spectroscopy for In-Field Detection of Pesticides: A Test on Dimethoate Residues in Water and on Olive Leaves
Source: Molecules. 2019 Jan 15;24(2):292. doi: 10.3390/molecules24020292 (PMC6359141; doi:10.3390/molecules24020292)
Supplement: Supplementary file 1 [file molecules-24-00292-s001.pdf]

Surface enhanced Raman spectroscopy for in-field detection of pesticides: A test on dimethoate residues in water and on olive leaves

L. Tognaccini[1], M. Ricci [1,2], C. Gellini [1], A. Feis[1], G. Smulevich[1,*] and M. Becucci[1,2,3,*]

[1] Department of Chemistry 'U. Schiff', Università degli Studi di Firenze, via della Lastruccia 3-13, 50019 Sesto Fiorentino (Fi), Italy
[2] Department of Photonics, St. Petersburg Electrotechnical University, Ul. Prof. Popova, St. Petersburg 197376, Russia
[3] EuropeanLaboratory for Non-Linear Spectroscopy—LENS, via N. Carrara 1, 50019 Sesto Fiorentino (Fi), Italy
* Corresponding authors: giulietta.smulevich@unifi.it; maurizio.becucci@unifi.it

Supplementary Information

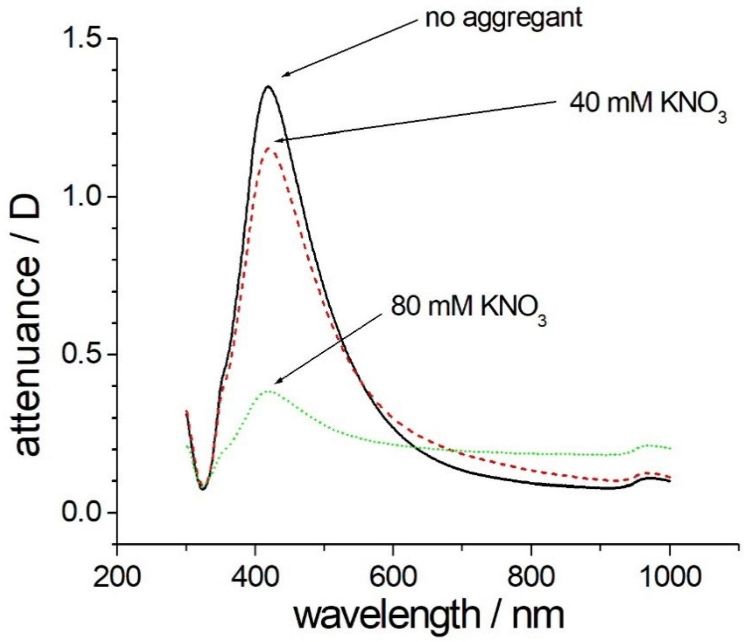

Figure S1. The attenuance spectrum (2 mm path-length) of the colloidal dispersion with AgNPs from synthesis and with different concentration of KNO_3 (aggregant).

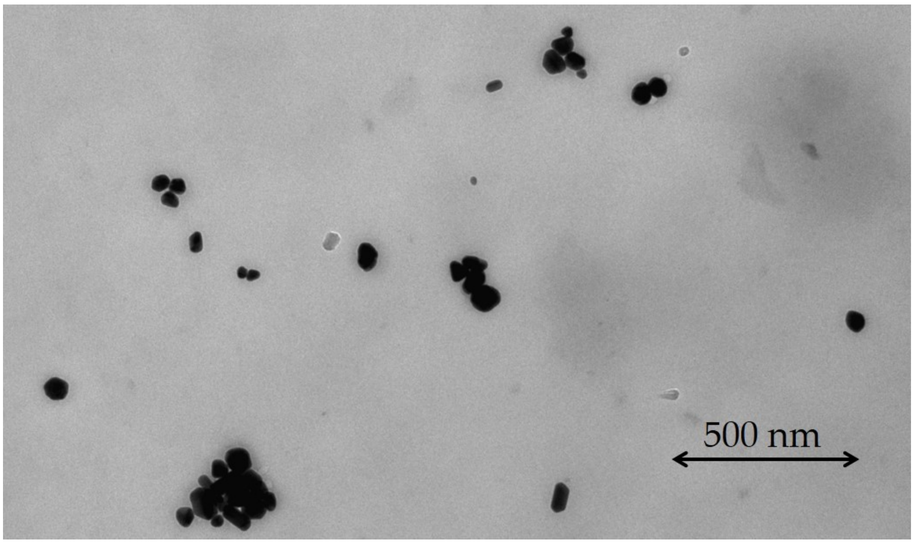

Figure S2. TEM image of partly aggregated AgNPs deposited on a clean surface.

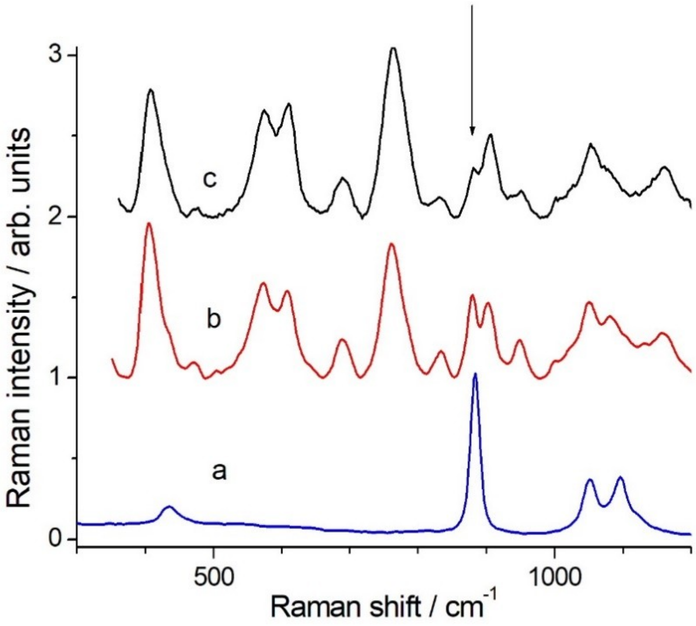

Figure S3. Raman spectrum of ethanol taken with the 785 nm excitation (a), and SERS spectra of 10^{-4}M DMT taken with the 1064 (b) and 785 nm (c) excitation, in presence of 10% ethanol. The arrow points to the ethanol strong band (880 cm^{-1}) used to evaluate the relative SERS enhancement factors. Spectra are baseline corrected.

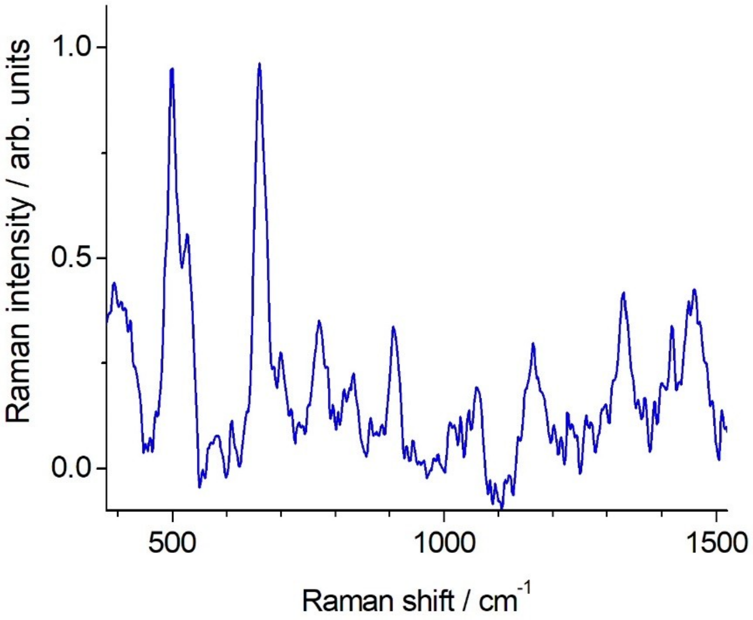

Figure S4. Raman spectrum of DMT 10^{-2} M in water (Bruker FT-Raman spectrometer, 1064nm, 300mW, 5000 averages, background subtracted)

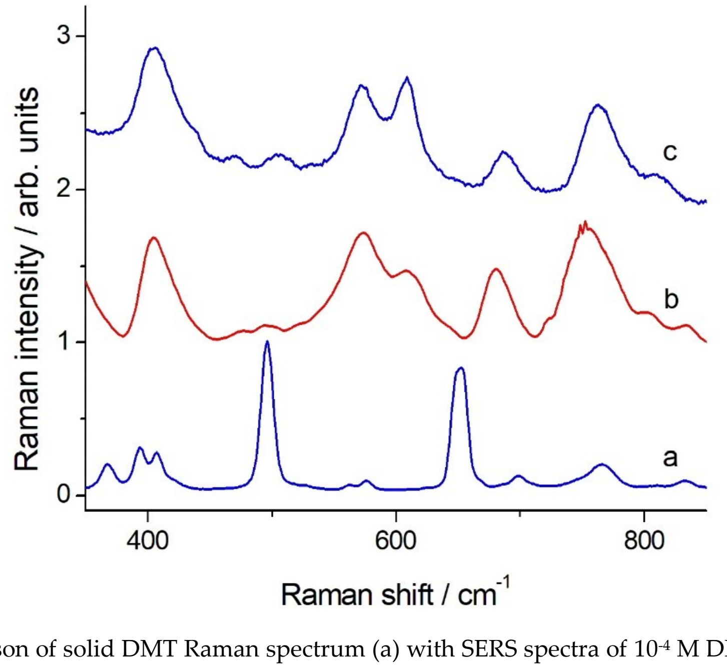

Figure S5. Comparison of solid DMT Raman spectrum (a) with SERS spectra of 10^{-4} M DMT (b) and of the same solution sprayed on a glass surface and dried (c). (a) and (c) measured on microRaman Renishaw RM2000 spectrometer;(b) measured on BWTek portable spectrometer.

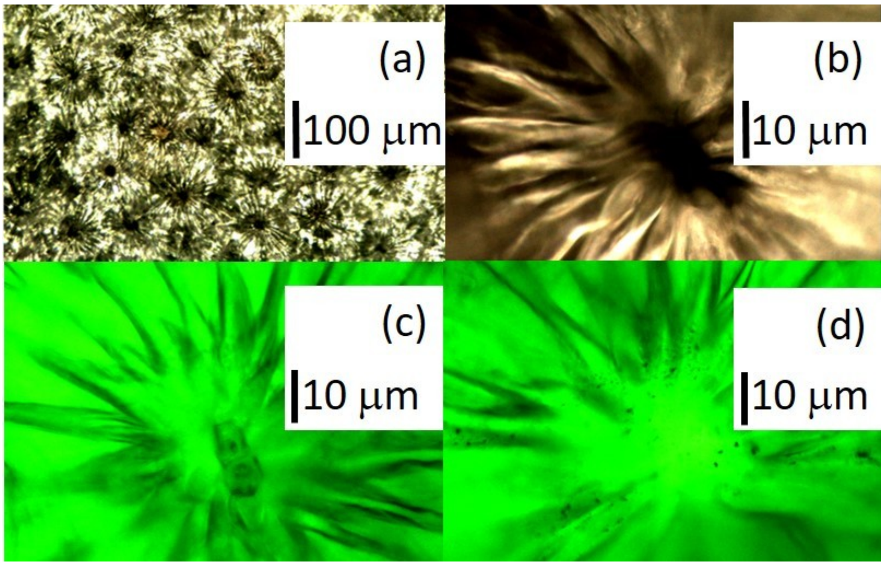

Figure S6. Microscope bright field images of clean and AgNPs treated olive leaves. (a), (b) clean olive leaf (back side) observed with 10x and 100x objectives, reflection mode; (c) clean leaf (back side), 100x objective, transmission mode; (d) olive leaf treated on the back side with aggregated AgNPs, 100x objective, transmission mode.

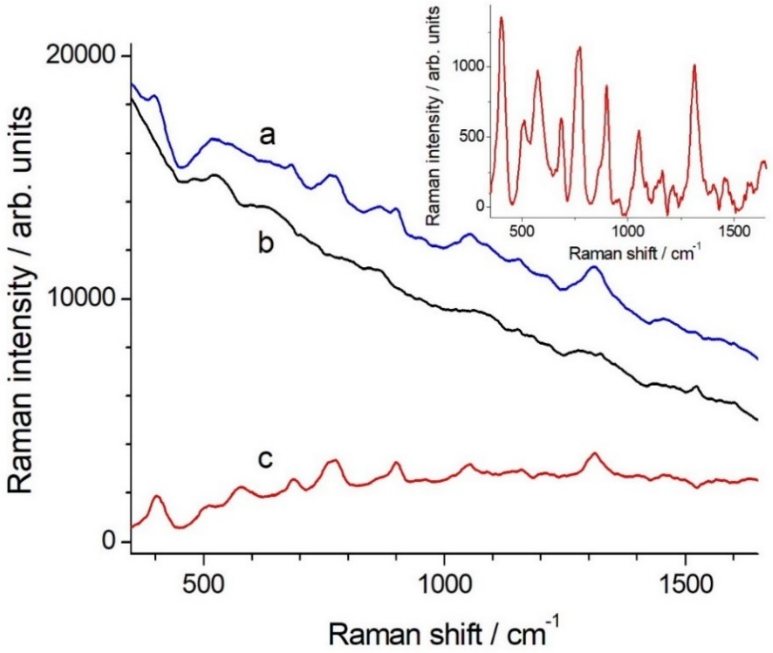

Figure S7. Measurements with the BWTek portable micro-Raman spectrometer: (a) SERS spectrum of the DMT treated area of an olive leaf (10^{-2} M DMT), (b) Raman spectrum of the clean leaf, and (c) their difference spectrum.Experimental details: 40x objective, 785 nm excitation wavelength, 2.5 mW laser power on the sample, 10 s integration time and 10 averages. The inset shows the difference spectrum (background subtracted) that in Figure 5 is compared to those obtained from DMT/OMT solutions.
